# A trans‐omics assessment of gene–gene interaction in early‐stage NSCLC

**DOI:** 10.1002/1878-0261.13345

**Published:** 2022-12-05

**Authors:** Jiajin Chen, Yunjie Song, Yi Li, Yongyue Wei, Sipeng Shen, Yang Zhao, Dongfang You, Li Su, Maria Moksnes Bjaanæs, Anna Karlsson, Maria Planck, Johan Staaf, Åslaug Helland, Manel Esteller, Hongbing Shen, David C. Christiani, Ruyang Zhang, Feng Chen

**Affiliations:** ^1^ Department of Biostatistics, Center for Global Health, School of Public Health Nanjing Medical University Nanjing China; ^2^ Department of Biostatistics University of Michigan Ann Arbor MI USA; ^3^ Department of Environmental Health Harvard T.H. Chan School of Public Health Boston MA USA; ^4^ China International Cooperation Center for Environment and Human Health Nanjing Medical University Nanjing China; ^5^ Pulmonary and Critical Care Division, Department of Medicine Massachusetts General Hospital and Harvard Medical School Boston MA USA; ^6^ Department of Cancer Genetics, Institute for Cancer Research Oslo University Hospital Oslo Norway; ^7^ Division of Oncology, Department of Clinical Sciences Lund and CREATE Health Strategic Center for Translational Cancer Research Lund University Lund Sweden; ^8^ Institute of Clinical Medicine University of Oslo Oslo Norway; ^9^ Josep Carreras Leukaemia Research Institute Barcelona Spain; ^10^ Centro de Investigacion Biomedica en Red Cancer Madrid Spain; ^11^ Institucio Catalana de Recerca i Estudis Avançats Barcelona Spain; ^12^ Physiological Sciences Department, School of Medicine and Health Sciences University of Barcelona Barcelona Spain; ^13^ Department of Epidemiology, School of Public Health Nanjing Medical University Nanjing China; ^14^ Jiangsu Key Lab of Cancer Biomarkers, Prevention and Treatment, Cancer Center, Collaborative Innovation Center for Cancer Personalized Medicine Nanjing Medical University Nanjing China; ^15^ State Key Laboratory of Reproductive Medicine Nanjing Medical University Nanjing China

**Keywords:** G × G interactions, NSCLC, overall survival, prognosis, trans‐omics

## Abstract

Epigenome‐wide gene–gene (G × G) interactions associated with non‐small‐cell lung cancer (NSCLC) survival may provide insights into molecular mechanisms and therapeutic targets. Hence, we proposed a three‐step analytic strategy to identify significant and robust G × G interactions that are relevant to NSCLC survival. In the first step, among 49 billion pairs of DNA methylation probes, we identified 175 775 G × G interactions with *P*_Bonferroni_ ≤ 0.05 in the discovery phase of epigenomic analysis; among them, 15 534 were confirmed with *P* ≤ 0.05 in the validation phase. In the second step, we further performed a functional validation for these G × G interactions at the gene expression level by way of a two‐phase (discovery and validation) transcriptomic analysis, and confirmed 25 significant G × G interactions enriched in the 6p21.33 and 6p22.1 regions. In the third step, we identified two G × G interactions using the trans‐omics analysis, which had significant (*P* ≤ 0.05) epigenetic *cis*‐regulation of transcription and robust G × G interactions at both the epigenetic and transcriptional levels. These interactions were cg14391855 × cg23937960 (*β*_interaction_ = 0.018, *P* = 1.87 × 10^−12^), which mapped to *RELA* × *HLA‐G* (*β*_interaction_ = 0.218, *P* = 8.82 × 10^−11^) and cg08872738 × cg27077312 (*β*_interaction_ = −0.010, *P* = 1.16 × 10^−11^), which mapped to *TUBA1B* × *TOMM40* (*β*_interaction_ =−0.250, *P* = 3.83 × 10^−10^). A trans‐omics mediation analysis revealed that 20.3% of epigenetic effects on NSCLC survival were significantly (*P* = 0.034) mediated through transcriptional expression. These statistically significant trans‐omics G × G interactions can also discriminate patients with high risk of mortality. In summary, we identified two G × G interactions at both the epigenetic and transcriptional levels, and our findings may provide potential clues for precision treatment of NSCLC.

## Graphical abstract

A three‐step, trans‐omics study identified two gene–gene interactions, cg14391855 × cg23937960 (mapped to *RELA* × *HLA‐G*) as well as cg08872738 × cg27077312 (mapped to *TUBA1B* × *TOMM40*), which were significantly and robustly associated with NSCLC survival at both the epigenetic and transcriptional levels. Our findings have implications of precision treatment by providing therapeutic targets for early‐stage NSCLC patients.

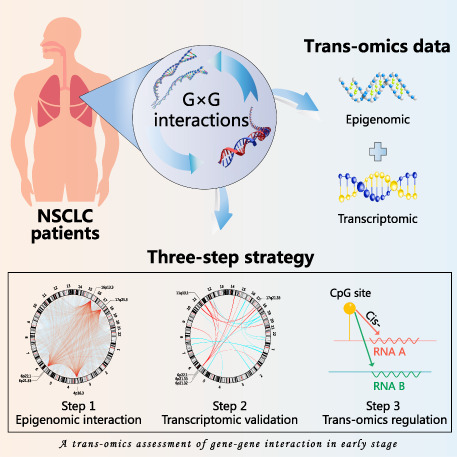

**Abbreviations**
G × G interaction: gene–gene interaction
GEO: Gene Expression Omnibus
GO: Gene Ontology
KEGG: Kyoto Encyclopedia of Genes and Genomes
LCSERG: Lung Cancer Survival Epigenome Research Group
LUAD: lung adenocarcinoma
LUSC: lung squamous cell carcinoma
MHC: major histocompatibility complex
NF‐κB: nuclear factor‐κB
NSCLC: non‐small‐cell lung cancer
QC: quality control
ROS: reactive oxygen species.
SD: standard deviation
TCGA: The Cancer Genome Atlas

## 1 Introduction

Lung cancer is widely prevalent and is the most lethal disease among all malignant cancers; in year 2020 alone, more than 2.2 million patients were diagnosed with lung cancer and nearly 1.8 million patients succumbed to the disease [1]. About 85% of lung cancer cases are non‐small‐cell lung cancer (NSCLC). Compared to those diagnosed with advanced‐stage NSCLC, early‐stage patients tend to have a more favourable prognosis. However, wide clinical variation is observed among early‐stage NSCLC patients, even among those with similar clinical characteristics [2], indicating possible heterogenous molecular characteristics of the disease [3].

DNA methylation, a heritable, reversible, and epigenetic modification involving the DNA spatial conformation [4], plays an essential role in prognosis and therapeutic target of cancers [5], including NSCLC [6]. Moreover, gene–gene (G × G) interactions may provide pivotal clues regarding the biologic mechanisms of complex diseases [7] and enhance the accuracy of prediction models [8, 9]. G × G interactions, as an essential element of personalized medicine, reflect that the effects of one gene on the disease outcome may vary across patients with different characteristics on another gene. Our previous studies have identified several epigenetic G × G interactions [10, 11] and gene–environment (G × E) interactions [12, 13, 14] relevant to NSCLC survival. However, these studies only focused on target genes that were identified in the literature. Subsequently, we performed the first genome‐wide G × G interaction study of lung cancer risk among the Asian and European populations, respectively [15, 16], and identified several novel biomarkers associated with lung cancer risk. We further conjectured that a comprehensive epigenomic G × G interaction study of NSCLC survival could identify novel interactions, providing insights into molecular mechanism and guiding precision treatment of NSCLC. However, virtually no studies have related epigenome‐wide G × G interactions to NSCLC survival, owing to enormous computational challenges and lack of reproducibility.

In this study, we integrated epigenomic and transcriptomic data of multiple cohorts and utilized a three‐step analytic strategy to identify robust G × G interactions. First, we performed an epigenome‐wide G × G interaction study of lung cancer survival using samples from Lung Cancer Survival Epigenome Research Group (LCSERG) and further validated the selected signals using The Cancer Genome Atlas (TCGA). Second, we functionally evaluated the significant epigenetic G × G interactions and validated them at the gene expression level using transcriptomic data. Third, focusing on these G × G interactions having epigenetic *cis*‐regulation of transcription, we conducted a trans‐omics mediation analysis.

## 2 Materials and methods

### 2.1 Study populations of DNA methylation data

We harmonized the DNA methylation data for early‐stage (stages I and II) NSCLC patients from LCSERG and TCGA. LCSERG is an international collaborative team composed of four study sites, including USA‐Harvard, Spain, Norway, and Sweden [9]. All patients provided written informed consent. The study methodologies conformed to the standards set by the Declaration of Helsinki and was approved by the local ethics committee.

#### 2.1.1 USA‐Harvard

The USA‐Harvard site consisted of patients recruited at Massachusetts General Hospital (MGH) since 1992 [17]. All were newly diagnosed and histologically confirmed as primary NSCLC at the time of recruitment. Snap‐frozen tumour samples were taken from patients during complete resection. A series of 151 early‐stage patients selected in this study had complete survival information. Tumour DNA was extracted from 5‐μm‐thick histopathological sections. Each specimen was evaluated by an MGH pathologist for the amount (tumour cellularity > 70%) and quality of tumour cells. All specimens were histologically classified using the Word Health Organization (WHO) criteria. The study was approved by the Institutional Review Boards of the Massachusetts General Hospital (Partners Human Research Committee, Protocol #1999P004935/MGH).

#### 2.1.2 Spain

The Spanish centre is a collaborative study centre, consisted of multiple research institutions from Spain (Catalan Institute of Oncology; Center for Applied Medical Research; and Bellvitge Biomedical Research Institute), Italy (IRCCS Foundation National Cancer Institute; and University of Turin), UK (University of Liverpool Cancer Research Centre), France (CHU Albert Michallon), and the USA (University of Michigan Medical School). Tumours were collected by surgical resection from 226 patients between 1991 and 2009 [18]. DNA extraction was performed on tumour specimens (10 μm‐thick, tumour cellularity > 50%). The study was approved by the Bellvitge Biomedical Research Institute Institutional Review Board (PR055/10).

#### 2.1.3 Norway

Participants were 133 lung adenocarcinoma (LUAD) patients with operable lung cancer tumours seen at the Oslo University Hospital between 2006 and 2011 [19]. Tumour tissues were collected during surgery, snap‐frozen in liquid nitrogen, and stored at −80 °C until DNA isolation. The project was approved by the Oslo University Institutional Review Board and the Regional Ethics Committee (S‐05307).

#### 2.1.4 Sweden

Tumour tissue samples were collected from 103 patients with early‐stage NSCLC who underwent operation, including 80 patients with LUAD and 23 patients with lung squamous cell carcinoma (LUSC) at the Skane University Hospital [20]. The study was approved by the Regional Ethical Review Board in Lund, Sweden (Registration nos. 2004/762 and 2008/702).

#### 2.1.5 TCGA

A total of 332 LUAD and 285 LUSC with full DNA methylation, survival time, and covariates data were included. Level 1 HumanMethylation450 DNA methylation data from patients with early‐stage NSCLC were downloaded from Genomic Data Commons Data Portal (GDC) resources.

### 2.2 Quality control for DNA methylation data

DNA methylation was assessed with Illumina Infinium HumanMethylation450 BeadChips (Illumina Inc., San Diego, CA, USA). Raw image data were imported into GenomeStudio Methylation Module V1.8 (Illumina Inc.) to calculate methylation signals and to perform normalization, background subtraction, and quality control (QC). Unqualified probes were excluded if meeting any of these exclusion criteria: (a) failed detection (*P* > 0.05) in 5% samples; (b) coefficient of variance < 5%; (c) methylated values of CpG probes were all 0 (unmethylated) or 1 (methylated) across all samples; (d) common single‐nucleotide polymorphisms located in probe sequence or in 10‐bp flanking regions; (e) cross‐reactive probes [21]; and (f) data did not pass QC in all centres. Methylation signals were further processed for quantile normalization as well as types I and II probe correction. Batch effects were adjusted according to the best pipeline and by a comparative study [22]. Details of the QC process are described in Fig. S1.

### 2.3 Study populations and quality control of gene expression data

Gene expression data for early‐stage NSCLC were derived from the Gene Expression Omnibus (GEO) and TCGA, and early‐stage NSCLC patients profiled by Affymetrix Human Genome U133 Plus 2.0 Array were retrieved from GEO (GSE10245, GSE29013, GSE31210, and GSE50081) (Table S1). Included in the discovery phase of transcriptomic analysis were 484 patients with available overall survival time, clinical stage, and other clinical covariates. Meanwhile, 613 early‐stage NSCLC patients were downloaded from GDC resources for validation of transcriptional G × G interactions. The TCGA workgroup completed the mRNA sequencing data processing and QC. Level 3 gene quantification data were downloaded from the TCGA data portal and were further checked for quality. Gene probes were excluded if the missing rate > 80%, and batch effects were corrected with ComBat. The expression value of each gene was transformed on a log_2_ scale and standardized.

### 2.4 Study design and statistical analysis

**Fig. 1:**
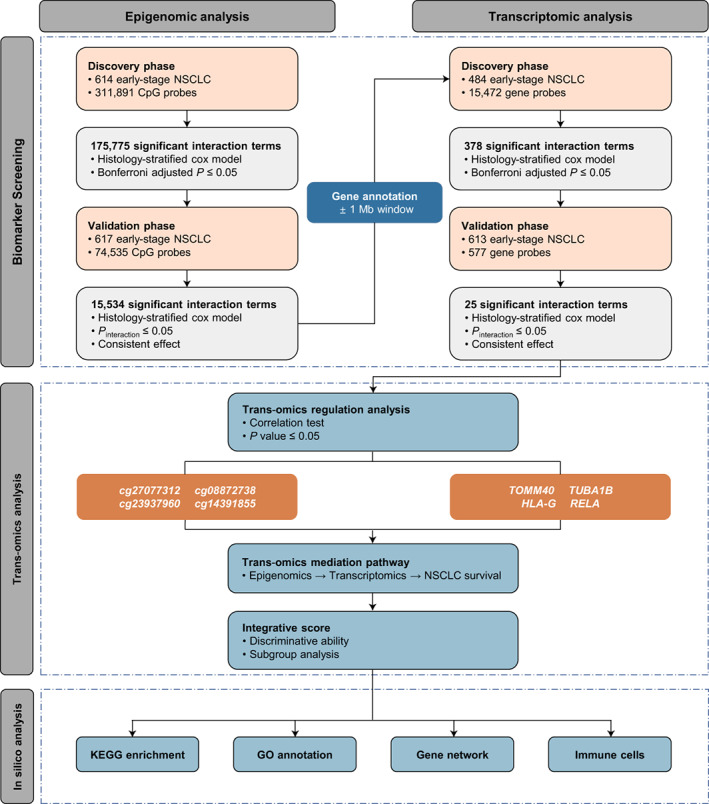
Flow chart of three‐step study design. In the epigenomic analysis step, we performed a two‐phase strategy to screen and validate epigenome‐wide G × G interactions. In the transcriptomic analysis step, we firstly conducted a gene annotation and again performed a two‐phase study to evaluate and validate those interactions at transcriptional level. In the trans‐omics analysis step, we explored the trans‐omics regulatory relationships and evaluated indirect effect of epigenetic G × G interactions on NSCLC survival mediated through transcriptional G × G interactions, and followed by a series of *in silico* analyses for potential biological functions.

Figure 1 depicts the workflow of the proposed three‐step analytic strategy. These three steps are detailed below.

#### 2.4.1 A two‐phase epigenome‐wide G × G interaction study

We conducted a two‐phase epigenomic study to screen G × G interactions out of a massive number of pairs. In the discovery phase, using the LCSERG dataset, we applied Cox proportional hazards models, adjusted by covariates (age, sex, smoking status, clinical stage, and study centre) and stratified by histology (see the Model below), to all G × G interactions one at a time, and identified significant G × G interactions among 49 billion pairs of DNA methylation probes. Significance was set to be at the level of 1.03 × 10^−12^ = 0.05/(311 891 × 311 890/2) by adjusting for multiple tests with the Bonferroni method. In the validation phase, we further confirmed the selected interactions using the TCGA cohort; only those with *P* ≤ 0.05 and with the same effect directions as in the discovery phase would be selected as the candidate G × G interactions and passed onto the next steps.

$$\text{Model}:h\left(t\right)=\begin{array}{l} h_{0}\left(t\right)\exp(\alpha_{1}\times G_{1}+\alpha_{2}\times G_{2}+\alpha_{3}\times G_{1}\\ \times G_{2}+\sum \beta_{i}\times {\text{covariate}}_{i}). \end{array}$$

#### 2.4.2 A two‐phase functional validation of G × G interactions in transcriptomic data

We evaluated the candidate G × G interactions at the transcriptional level, by annotating genes in the amplified regions within a 1 Mb window upstream and downstream for each gene. We applied a two‐phase strategy, similar to that outlined in Section 2.4.1, to screen and validate significant G × G interactions at the transcriptional level. Selected as the final candidate G × G interactions would be those with a *P*_Bonferroni_ ≤ 0.05 in the discovery phase based on the GEO dataset, with *P* ≤ 0.05 in the validation phase based on the TCGA dataset, and with the same effect directions in these two datasets.

#### 2.4.3 Trans‐omics regulation and mediation analysis

For G × G interactions with significant effects at both the epigenetic and transcriptional levels, we evaluated the trans‐omics regulation between the DNA methylations and gene expressions via the Spearman correlation. The DNA methylation probes located within 1 Mb distances upstream or downstream of its gene, and meanwhile significantly correlated with gene expression were defined as these having significant epigenetic *cis*‐regulation of transcription. Then, G × G interactions with significant epigenetic *cis*‐regulation of transcription were identified to be robust interactions. Moreover, we performed a trans‐omics mediation analysis to evaluate whether the prognostic effect of epigenetic G × G interactions on NSCLC survival was mediated through affecting the corresponding transcriptional G × G interactions, implementing by VanderWeele's method [23].

#### 2.4.4 Statistical analysis

Continuous variables were summarized as mean ± standard deviation (SD), and categorized variables were described by frequency (*n*) and proportion (%). Kaplan–Meier survival curves illustrated the survival differences across different risk groups. The histology‐stratified Cox proportional hazards models, adjusted for age, sex, smoking status, clinical stage, and study centre, were used to model the adjusted effect of each interaction. The epigenetic score and transcriptional score were defined as a linear combination of G × G interactions of two omics, respectively, with coefficients as weights derived from the multivariable Cox proportional hazards models. We applied Gene Ontology (GO) annotation and Kyoto Encyclopedia of Genes and Genomes (KEGG) pathway enrichment analyses to evaluate potential biological functions of screened biomarkers, and used gene network analysis to explore the relationship between screened genes and immune checkpoints by implementing GeneMANIA [24]. Proportions of immune cells were inferred by using CIBERSORT [25].

Statistical analyses were performed using r version 3.6.3 (The R Foundation of Statistical Computing, Vienna, Austria).

## 3 Results

### 3.1 Sample characteristics of the study population

After QC, included in this study were 1230 early‐stage NSCLC patients with DNA methylation data and 1097 patients with gene expression data. The demographic and clinical information of these patients are detailed in Table 1 and Table S2.

**Table 1:** Demographic and clinical descriptions for early‐stage NSCLC patients with DNA methylation data in five international study centres. LUAD, lung adenocarcinoma; LUSC, lung squamous cell carcinoma.

Variables	Discovery phase					Validation phase	Combined dataset
	USA‐Harvard (*N* = 151)	Spain^a^ (*N* = 226)	Norway (*N* = 133)	Sweden (*N* = 103)	All (*N* = 613)	USA‐TCGA (*N* = 617)	Overall (*N* = 1230)
Age (years)	67.67 ± 9.92	65.67 ± 10.58	65.52 ± 9.34	67.54 ± 9.99	66.44 ± 10.08	66.51 ± 9.47	66.48 ± 9.78
Sex							
Female	67 (44.37)	105 (46.46)	71 (53.38)	54 (52.43)	297 (48.45)	255 (41.33)	552 (44.88)
Male	84 (55.63)	121 (53.54)	62 (46.62)	49 (47.57)	316 (51.55)	362 (58.67)	678 (55.12)
Smoking status							
Never	18 (11.92)	30 (13.57)	17 (12.78)	18 (17.47)	83 (13.65)	55 (9.18)	138 (11.43)
Former	81 (53.64)	120 (54.30)	74 (55.64)	54 (52.43)	329 (54.11)	376 (62.77)	705 (58.41)
Current	52 (34.44)	71 (32.13)	42 (31.58)	31 (30.10)	196 (32.24)	168 (28.05)	364 (30.16)
Unknown	0	5	0	0	5	18	23
Clinical stage							
I	104 (68.87)	183 (80.97)	93 (69.92)	95 (92.23)	475 (77.49)	393 (63.70)	868 (70.57)
II	47 (31.13)	43 (19.03)	40 (30.08)	8 (7.77)	138 (22.51)	224 (36.30)	362 (29.43)
Histology							
LUAD	96 (63.58)	183 (80.97)	133 (100.00)	80 (77.67)	492 (80.26)	332 (53.81)	824 (66.99)
LUSC	55 (36.42)	43 (19.03)	0 (0.00)	23 (22.33)	121 (19.74)	285 (46.19)	406 (33.01)
Chemotherapy							
No	142 (94.04)	177 (90.77)	102 (76.69)	67 (90.54)	488 (88.25)	1974 (76.98)	682 (84.72)
Yes	9 (5.96)	18 (9.23)	31 (23.31)	7 (9.46)	64 (11.75)	58 (23.02)	123 (15.28)
Unknown	0	31	0	29	60	365	425
Radiotherapy							
No	132 (87.42)	184 (94.36)	132 (99.25)	74 (100.00)	522 (94.39)	239 (94.84)	761 (94.53)
Yes	19 (12.58)	11 (5.64)	1 (0.75)	0 (0.00)	31 (5.61)	13 (5.16)	44 (5.47)
Unknown	0	31	0	29	60	365	425
Adjuvant therapy^b^							
No	127 (84.11)	168 (86.15)	101 (75.94)	67 (90.54)	463 (83.73)	187 (74.21)	650 (80.75)
Yes	24 (15.89)	27 (13.85)	32 (24.06)	7 (9.46)	90 (16.27)	65 (25.79)	155 (19.25)
Unknown	0	31	0	29	60	365	425
Survival year							
Median survival	6.66 (5.41, 7.87)	7.12 (5.06, 9.63)	7.36 (6.77, 7.95)^c^	7.39 (4.98, 9.12)	7.39 (6.50, 8.23)	4.54 (3.68, 5.41)	6.60 (5.84, 7.35)
Died (%)	122 (80.79)	101 (44.69)	42 (31.58)	58 (56.31)	323 (52.69)	142 (23.01)	465 (37.80)

^a^ The Spanish centre is a collaborative study centre with samples recruited from Spain, Italy, UK, France, and the USA.

^b^ Including chemotherapy or radiotherapy.

^c^ The restricted mean survival time was given since the median is not available.

### 3.2 Two robust G × G interactions identified in epigenome‐wide G × G interaction study and trans‐omics validation in transcriptome

**Fig. 2:**
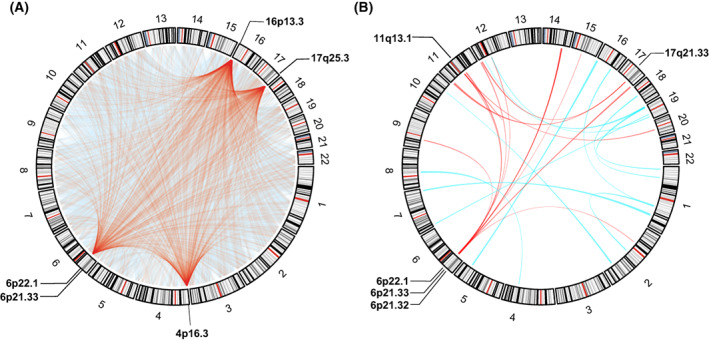
The circos plot for visualization of G × G interactions. Results of G × G interactions were given at epigenetic level (A) and transcriptional level (B), respectively. The chords represent interactions exists between two linked chromosome segments, and the chords of the top five enriched regions are highlighted in red.

Using the LCSERG cohort in the discovery phase of the epigenomic analysis, we identified a total of 175 775 epigenetic G × G interactions (*P*_Bonferroni_ ≤  0.05) significantly associated with NSCLC survival. Among them, 15 534 interactions remained significant (*P* ≤ 0.05) in the validation phase based on the TCGA cohort (Table S3). These interactions were enriched in the 16p13.3, 4p16.3, 6p21.33, 17q25.3, and 6p22.1 regions (Fig. 2A), and further gene annotation for them resulted in 6 850 451 epigenetic *cis*‐regulatory gene pairs. By way of transcriptomic analysis, a total of 378 transcriptional G × G interactions were found to be significantly associated with NSCLC survival in the discovery phase; of them, 25 interactions were successfully validated in the validation phase (Table S4), and the 6p21.32, 6p21.33, 6p22.1, 11q13.1, and 17q21.33 regions were identified to be the enriched functional regions (Fig. 2B).

Among the interactions deemed significant at both the epigenetic and transcriptional levels, we evaluated the trans‐omics *cis*‐regulatory relationship between DNA methylations and their mapped gene expressions, and found that two G × G interactions had significant trans‐omics regulations (*r*_cg14391855‐*RELA*_ = −0.11, *P* = 6.80× 10^−3^, *r*_cg23937960‐*HLA‐G*_ = −0.08, *P* = 3.86 × 10^−2^; and *r*_cg08872738‐*TUBA1B*_ = 0.12, *P* = 4.10 × 10^−3^, *r*_cg27077312‐*TOMM40*_ = 0.15, *P* = 1.87 × 10^−4^) (Table S5). Moreover, these G × G interactions remained significant in the subgroup defined by histology, except for a subgroup that have only 73 LUSC samples with gene expression, which might be due to a very limited sample size. Nevertheless, heterogeneity test suggested no significant heterogenous effect was observed between LUAD and LUSC (Fig. S2). Therefore, cg14391855 × cg23937960 (mapped to *RELA* × *HLA‐G*) as well as cg08872738 × cg27077312 (mapped to *TUBA1B* × *TOMM40*) were viewed as the robust interactions, which were passed onto subsequent analyses.

### 3.3 Interaction patterns and effect modifications of two G × G interactions on NSCLC survival

**Fig. 3:**
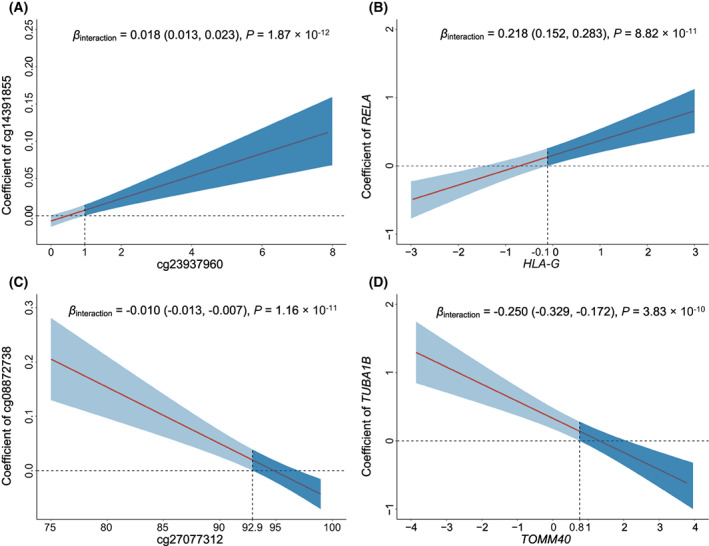
Interaction patterns of the screened G × G interactions. Interactions of cg14391855 × cg23937960 (A), which mapped to *RELA* × *HLA‐G* (B); as well as cg08872738 × cg27077312 (C), which mapped to *TUBA1B* × *TOMM40* (D) were displayed as the effects of one gene (*y* axis) varied by the different level of another gene (*x* axis). Coefficients, 95% CIs, and *P* values were derived from histology‐stratified Cox proportional hazards models adjusted for age, sex, smoking status, clinical stage, and study centre. The shaded area represents 95% confidence interval. The horizontal dashed line indicates the zero correlation. The vertical dashed line represents the intersection of the lower bound of the 95% CI and the zero correlation.

Significant synergistic interactions were observed between cg14391855 and cg23937960 (*β*_interaction_ = 0.018, 95% CI: 0.013–0.023, *P* = 1.87 × 10^−12^) (Fig. 3A), which were mapped to *RELA* × *HLA‐G* (*β*_interaction_ = 0.218, 95% CI: 0.152–0.283, *P* = 8.82 × 10^−11^) (Fig. 3B). In contrast, as the methylation level of cg27077312 increased, the effect of cg08872738 decreased (*β*_interaction_ = −0.010, 95% CI: −0.013 to −0.007, *P* = 1.16 × 10^−11^) (Fig. 3C). Moreover, the antagonistic interaction between their mapped genes, *TOMM40* × *TUBA1B*, was identified (*β*_interaction_ = −0.250, 95% CI: −0.329 to −0.172, *P* = 3.83 × 10^−10^) (Fig. 3D).

**Fig. 4:**
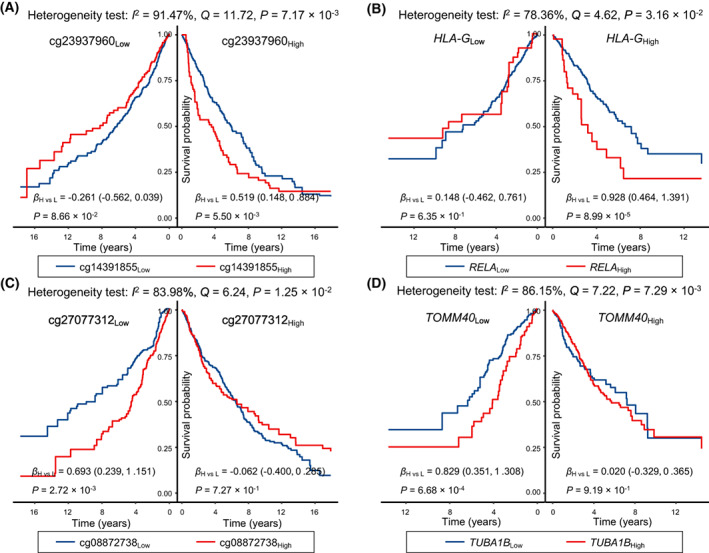
Kaplan–Meier survival curves for effect modifications. Survival differences of cg14391855 stratified by cg23937960 (*N*_Low_ = 615, *N*_High_ = 616) (A), *RELA* stratified by *HLA‐G* (*N*_Low_ = 548, *N*_High_ = 549) (B), cg08872738 stratified by cg27077312 (*N*_Low_ = 615, *N*_High_ = 616) (C), and *TUBA1B* stratified by *TOMM40* (*N*_Low_ = 548, *N*_High_ = 549) (D), respectively. The coefficients, 95% CIs, and *P* values for each strata were derived from histology‐stratified Cox proportional hazards models adjusted for age, sex, smoking status, clinical stage and study centre. Heterogeneity tests were used to evaluate effect modifications.

To explore the effect modifications, we evaluated the effects of cg14391855 among patients with low and high levels of cg23937960. We observed a harmful effect of cg14391855 in patients with high methylation level of cg23937960 (*β*_H vs L_ = 0.519, 95% CI: 0.148–0.884, *P* = 5.50 × 10^−3^), but did not note any significant effects of cg14391855 among those with low methylation level of cg23937960 (*β*_H vs L_ = −0.261, 95% CI: −0.562 to 0.039, *P* = 0.087), indicating a significant heterogeneity of the effects of cg14391855 (*I* ^2^ = 91.47%, *P* = 7.17 × 10^−3^); see Fig. 4A. We also observed heterogenous effects of *RELA* across patients with low and high expression levels of *HLA‐G* (*I* ^2^ = 78.36%, *P* = 3.16 × 10^−2^). Specifically, high *RELA* expression was associated with high mortality among patients with high *HLA‐G* gene expression (*β*_H vs L_ = 0.928, 95% CI: 0.464–1.391, *P* = 8.99 × 10^−5^), but not so among those with low *HLA‐G* gene expression (*β*_H vs L_ = 0.148, 95% CI: −0.462 to 0.761, *P* = 0.64) (Fig. 4B). Additionally, heterogeneity tests suggested significantly differential effects of cg08872738 across patients with different levels of cg27077312 (*β*_H vs L_ = 0.693, 95% CI: 0.239–1.151, *P* = 2.72 × 10^−3^ for low cg27077312 patients; *β*_H vs L_ = −0.062, 95% CI: −0.400 to 0.285, *P* = 0.73 for high cg27077312 patients; and *I* ^2^ = 83.98%, *P* = 1.25 × 10^−2^), and significantly differential effects of *TUBA1B* across patients with low and high *TOMM40* expression levels (*β*_H vs L_ = 0.829, 95% CI: 0.351–1.308, *P* = 6.68 × 10^−4^ for low *TOMM40* patients; *β*_H vs L_ = 0.020, 95% CI: −0.329 to 0.365, *P* = 0.92 for high *TOMM40* patients; and *I* ^2^ = 86.15%, *P* = 7.29 × 10^−3^).

### 3.4 Trans‐omics analysis of two G × G interactions

**Fig. 5:**
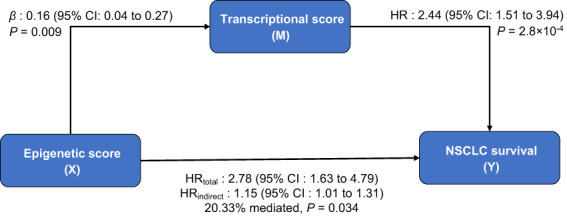
Mediation analysis for trans‐omics regulatory pathway. VanderWeele causal mediation analysis was conducted to evaluate the indirect effect, 95% CI, and *P* value of epigenetic G × G interactions on overall survival of NSCLC patients via transcriptional G × G interactions.

A significant trans‐omics regulation was observed between the epigenetic score and transcriptional score (*β* = 0.16, 95% CI: 0.04–0.27, *P* = 0.009). Further, mediation analysis for the trans‐omics pathway revealed that 20.3% of the effect of the epigenetic score on NSCLC survival were mediated via the transcriptional score (HR_indirect_ = 1.15, 95% CI: 1.01–1.31, *P* = 0.034) (Fig. 5).

**Fig. 6:**
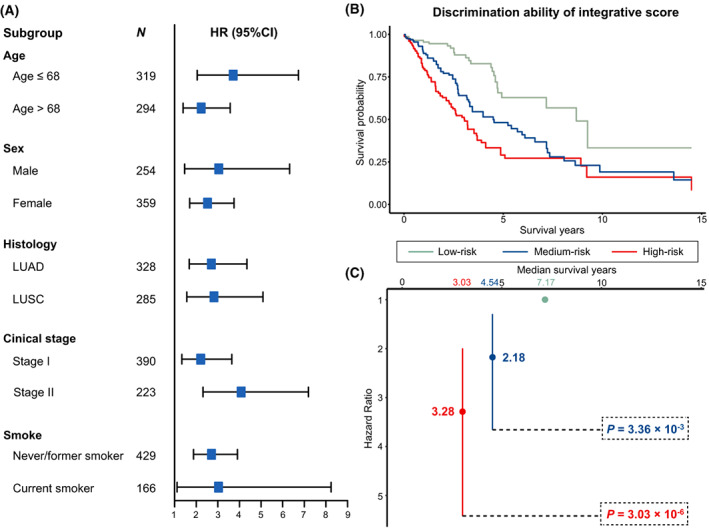
Subgroup analysis and discrimination ability of integrative score. (A) Forest plot for subgroup analysis of integrative score stratified by age, sex, histology, clinical stage, and smoke. (B) Discriminative ability of the integrative score, low‐ (*N* = 204), medium‐ (*N* = 205), and high‐risk group (*N* = 204) were defined by tertiles of integrative score. (C) The hazard ratios (HRs) for patients at medium‐ and high‐risk groups when comparing with patients at low risk. Significances were derived from histology‐stratified Cox proportional hazards models adjusted for age, sex, smoking status, clinical stage, and study centre.

We constructed an integrative score by linearly combining the epigenetic and transcriptional scores, with coefficients as weights derived from a multivariable Cox regression model, and found it robustly associated with NSCLC survival in patient subgroups defined by various covariates (Fig. 6A). To demonstrate the discriminative ability of this integrative score, we categorized patients into three subgroups based on the tertiles of the score and detected a dose–response association; higher‐percentile groups were associated with higher mortality (HR_M vs L_ = 2.18, 95% CI: 1.29–3.66, *P* = 3.36 × 10^−3^; HR_H vs L_ = 3.28, 95% CI: 1.99–5.42, *P* = 3.03 × 10^−6^) (Fig. 6B,C). Moreover, we noted that the discriminative ability of integrative score outperforms the clinical factors (Fig. S3).

### 3.5 *In silico* analyses for potential biological functions

**Fig. 7:**
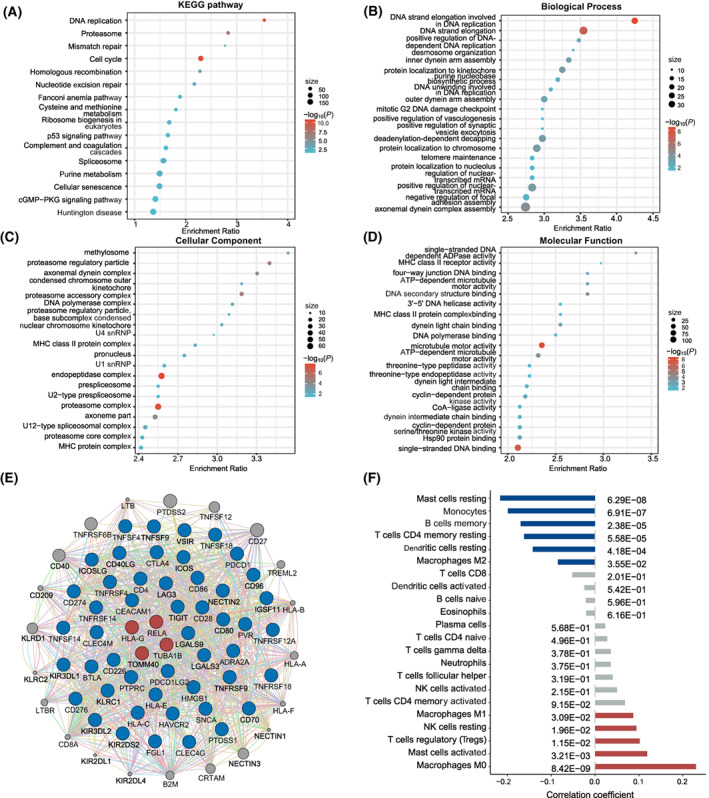
*In silico* analyses for potential biological functions of screened interactions. The functional enrichment analyses for co‐expressed genes of integrative score. (A) 16 significant KEGG pathways. (B) Top 20 significant biological process pathways. (C) Top 20 significant cellular component pathways. (D) Top 20 significant molecular function pathways. (E) Gene network of screened genes and immune checkpoint genes, which constructed by GeneMINIA. The blue circles represents the first neighbours of the screened genes, which suggests direct functional connections. (F) Correlations between proportions of immune cells and integrative score.

To explore the potential biological functions, we scanned the transcriptome‐wide gene expression probes to find those correlated with the genes included in the integrative score. As a result, we identified a total of 4588 co‐expressed genes, which were significantly enriched in 16 KEGG pathways, suggesting functions in cancer prognosis (Fig. 7A). Additionally, GO annotation analysis identified 491 biological process pathways, 78 molecular function pathways, and 141 cellular component pathways, which comprised the main activity of the major histocompatibility complex (MHC) (Fig. 7B–D). The gene network revealed the potential functional connections between the four genes with interactions and immune checkpoint genes (Fig. 7E). Therefore, we further inferred the proportions of immune cells using CIBERSORT and observed significant and positive correlations between the integrative score and five immune cells (e.g., with macrophages M0, *r* = 0.23, *P* = 8.42 × 10^−9^), as well as significant and negative correlations with 6 immune cells (e.g., with mast cells resting, *r* = −0.22, *P* = 6.29 × 10^−8^) (Fig. 7F). Additionally, numerous drugs targeting these interactions are documented in the DrugBank database (Table S6).

## 4 Discussion

Wide heterogeneity exists in the outcome of NSCLC patients, especially among the early‐stage NSCLC patients, and highlights the importance of personalized treatment [26], which refers to select specific treatment for patients based on their specific molecular biomarkers, to maximize the benefit from the treatments [27]. Gene–gene (G × G) interactions, in particular, at the epigenome level, play important roles in cancer progression and is an essential component of personalized treatment [26]. However, computational intensiveness and lack of reproducibility in G × G interaction analyses, in particular on the whole epigenome‐wide scale, impede their wide applications and hinder our ability to identify novel biomarkers at both the epigenome and transcriptome level. Our study addressed the serious challenges by using high performance computing clusters and proposing a 3‐step analytical strategy to identify epigenome‐wide G × G interactions, followed by a trans‐omics validation in transcriptome, and *in silico* analyses for exploring the biological functions, which might be a general schema that could be applied to other cancers.

To our knowledge, our work was the first attempt to identify epigenome‐wide G × G interactions and perform functional validation in transcriptome. Our study identified 16p13.3, 4p16.3, 6p21.33, 17q25.3, and 6p22.1 as the top five functional regions with largest number of epigenetic interactions. Among them, 6p21.33 and 6p22.1 were validated as functional enrichment regions in the transcriptomic interaction analysis. These two are well known regions, which house major histocompatibility complex (MHC) genes, including *HLA‐B*, *HLA‐C*, etc. in 6p21.33 and *HLA‐A*, *HLA‐E*, *HLA‐F*, *HLA‐G*, etc. in 6p22.1, respectively [28]. MHC molecules play essential roles in the immune system, antigen presentation [29]. Much evidence has linked MHC to the development, progression and prognosis of tumours [30], including NSCLC [31].

*RELA*, an encoder of the essential subunit of nuclear factor‐κB (NF‐κB), is involved in inflammation, immunity, tumourigenesis, and apoptosis, all of which are related to the progress and prognosis of tumour [32, 33]. NF‐κB can promote tumour survival via modifying apoptosis, cause inflammatory microenvironment by interacting with interleukins, and affect NSCLC survival [34]. Meanwhile, *HLA‐G*, a member of the MHC class I, is a well‐established immune checkpoint that plays an important regulatory role in tumour immune response [35, 36]. *HLA‐G* can inhibit the functions of NK and T cells, suppress the immune response, help tumour cells escape immune surveillance, and lead to poor prognosis of NSCLC patients [37]. Notably, the immune escape would further deteriorate inflammatory response [38], and increase the mortality risk from NF‐κB, leading to a synergistic effect [39]. Moreover, the protein product of *TUBA1B* is the main component of microtubules, which is involved in cell movement and intracellular trafficking, and affects cancer prognosis [40, 41]. Microtubules are involved in the mitochondrial motility under the hypoxic tumour microenvironment, promoting the perinuclear aggregation of mitochondria and production of reactive oxygen species (ROS) [42], and leading to cell damage, inflammation storm, and poor prognosis [43]. Additionally, *TOMM40*, which encodes a channel‐forming subunit of the translocase of the mitochondrial outer membrane, is an essential mediator of mitochondrial functions [44], and highly expressed *TOMM40* inhibits the generation of ROS [45, 46], causing an antagonistic interaction.

Another major contribution of our work is the proposed three‐step, multicohort analytic strategy with rigorous validation using independent cohorts and trans‐omics data. The alterations of DNA methylation regulate gene expression, thereby affecting the development, progress, and prognosis of diseases [47]. We integrated trans‐omics data for selecting G × G that were significant at both the epigenetic and transcriptional levels. Additionally, we developed an integrative score based on these interactions, which enabled us to identify patients with high mortality risk. Furthermore, the score was found associated with proportions of immune cell types, including mast cells, monocytes, B cells, T cells CD4, dendritic cells, NK cells and macrophages. The gene network analysis also indicated potential functional connections between the identified G × G interactions and the known immune checkpoint genes. The results, which may hint at the drug targets, have values in clinical immunotherapy and provide hypotheses for clinical trials.

Our study has several strengths. First, we addressed the computational burden and provided a computationally feasible landscape of analysing epigenetic interactions on NSCLC survival. Second, we used a strict strategy to control the false positives, which required G × G interactions to have a Bonferroni‐adjusted *P* value ≤ 0.05 in the discovery phase, retain significance (*P* ≤ 0.05) in the validation phase, and have the same effect directions in the two phases. Third, for reproducibility, we explored trans‐omics validation and regulatory relationships, which enhanced robustness. Finally, for clinical usage, we constructed an integrative score which can identify patients with high mortality risk, and the *in silico* analyses indicated the potential roles of the score in the immune response.

We acknowledge limitations. First, the majority of study subjects were Caucasian, which may limit the generalization of our results to the other ethnicity populations. Second, we only focused on *cis*‐regulatory genes within 1 Mb windows of CpGs in the trans‐omics validation stage, because *cis*‐regulations were considered to be causally and biologically interpretable [48, 49]. However, *trans*‐regulatory genes may also play important roles in the causal paths. Third, we used histology‐stratified Cox proportional hazards models to identify G × G interactions, which guaranteed the statistical power and also accounted for the heterogeneity between LUAD and LUSC. However, such pooled analysis may lose some histology‐specific signals. Finally, though our results were validated using various trans‐omics data, more biological experiments are warranted.

## 5 Conclusion

We identified two G × G interactions, cg14391855 ×cg23937960 (mapped to *RELA* × *HLA‐G*) as well as cg08872738 × cg27077312 (mapped to *TUBA1B* ×*TOMM40*), which were significantly and robustly associated with NSCLC survival at both the epigenetic and transcriptional levels. Our findings have implications of precision treatment by providing therapeutic targets for early‐stage NSCLC patients.

## Conflict of interest

The authors declare no conflict of interest.

## Author contributions

JC, YS, HS, DCC, RZ and FC contributed to the study design; SS, LS, MMB, AK, MP, JS, ÅH, ME, RZ and DCC involved in the data collection and quality control; JC, YS, YL, YW, SS, YZ, DY, DCC, RZ and FC involved in the analyses and interpretation; JC and YS involved in drafting the manuscript; YL, HS, DCC, RZ and FC revised the manuscript; all authors read and approved the final manuscript.

### Peer Review

The peer review history for this article is available at https://publons.com/publon/10.1002/1878‐0261.13345.

## Supporting information


**Fig. S1.** Quality control procedures for epigenome‐wide DNA methylation chip data from the USA (Harvard), Spain, Norway, and Sweden centres, as well as TCGA.
**Fig. S2.** Subgroup analysis of the identified G × G interactions in LUAD and LUSC.
**Fig. S3**. ROC plot for discrimination ability of integrative score and clinical covariates.
**Table S1.** Gene expression data downloaded from the GEO database.
**Table S2.** Demographic and clinical descriptions for early‐stage NSCLC patients with gene expression information from GEO and TCGA.
**Table S3.** Association results of 15,534 pairs of G × G interactions in the epigenomic analysis.
**Table S4.** Association results of 25 pairs of G × G interactions in the transcriptomic analysis.
**Table S5.** Correlation between DNA methylation probes and corresponding gene expressions.
**Table S6.** Potential targeted drug for screened G × G interactions derived from DrugBank database.

## Data Availability

The DNA methylation image data of USA‐Harvard, Spain, Norway and Sweden study cohort can be requested from DCC, ME, ÅH, and JS, respectively. Alternatively, it can be retrieved from GEO database (GSE39279, GSE66836 and GSE56044). Gene expression data were retrieved from GEO database (GSE10245, GSE29013, GSE31210, and GSE50081). TCGA: https://tcga‐data.nci.nih.gov; now hosted at GDC: https://portal.gdc.cancer.gov. GEO: https://www.ncbi.nlm.nih.gov/gds/.
